# An Interactive Mobile Phone App (SMART 5-A-DAY) for Increasing Knowledge of and Adherence to Fruit and Vegetable Recommendations: Development and Pilot Randomized Controlled Trial

**DOI:** 10.2196/14380

**Published:** 2019-11-20

**Authors:** Katherine Marie Appleton, David Passmore, Isobel Burn, Hanna Pidgeon, Philippa Nation, Charlotte Boobyer, Nan Jiang

**Affiliations:** 1 Bournemouth University Poole United Kingdom

**Keywords:** fruit, vegetables, diet therapy, knowledge, questionnaires, portion sizes

## Abstract

**Background:**

Fruit and vegetable consumption is important for health, but many individuals fail to consume adequate amounts for health benefits. Although many individuals are aware of current fruit and vegetable consumption recommendations, research suggests that adherence to these is hampered by low knowledge of the details of these recommendations.

**Objective:**

This paper reports the development and details of a pilot randomized controlled test of a novel interactive mobile phone app for addressing low knowledge of the UK 5-a-day fruit and vegetable recommendations.

**Methods:**

Requirements for the app were first defined by researchers and potential end users and prioritized using the MoSCoW (Must have, Should have, Could have, Won’t have) method. Second, a prototype mobile phone app was developed using an agile approach. Third, the prototype app was tested in a randomized controlled pilot trial for impacts on knowledge and intake of fruit and vegetables. Volunteers were randomized to either receive (n=50) or not receive the app (n=44) for 2 or 4 weeks, and fruit and vegetable knowledge, intake, and behavior were assessed at the beginning of the study and after 1 and 2 weeks or after 2 and 4 weeks, respectively. App usage and qualitative feedback were also investigated. All findings then informed the development of a final app.

**Results:**

Low knowledge of consumption recommendations centered around portion sizes and the need for variety, and an interactive mobile phone app was considered a suitable tool for improving this knowledge in a practical manner that would be available both at time of consumption and outside of these times. The pilot test revealed improved behavior after 2 weeks compared with baseline in volunteers who received the app, but improvements in knowledge on fruit and vegetable recommendations were found in both groups, and no improvements in fruit and vegetable intakes were found in formal measures. Patterns of app usage and qualitative feedback also suggested a number of modifications. The resultant final app incorporates several behavior change techniques (goal-setting, self-monitoring, and personalized feedback) as well as aiming to improve knowledge.

**Conclusions:**

A novel interactive mobile phone app was successfully developed based on requirements, and when tested in a pilot randomized controlled trial, this app was found to have some impacts on fruit and vegetable outcomes. Although benefits from the app were small, impacts will likely increase as a result of recent modifications. The final SMART 5-A-DAY app is available in the Google Play Store and now needs testing in the target population.

**Trial Registration:**

ClinicalTrials.gov NCT02779491; https://www.clinicaltrials.gov/ct2/show/NCT02779491

## Introduction

### Background

A high consumption of fruit(s) and vegetable(s) (FV) is associated with reduced risk of a number of global health concerns [1-10]. Resulting from these health benefits, the World Health Organization currently recommends consumption of at least 400 g FV per day [3-6], and governments around the world have operationalized these recommendations as recommended consumption of a number of portions of FV per day. Campaigns promoting these FV recommendations are easily available, but despite the campaigns, population FV intakes in Europe, the United States, and across the world remain low [11-14].

Populations do seem largely aware of FV consumption recommendations [15-21], and awareness of the recommendations has been associated with improved FV consumption [15,16,18,22,23]. Difficulties are reported, however, with the details of the recommendations. Consumers report confusion and poor knowledge around the foods that can be included as FV [24,25]; the amount of FV required in portion sizes [24-28], or contributing to portion sizes when portion sizes may be small, for example, for small fruits or in composite dishes [25]; the number of portions needed per day [28]; the need for a variety of FV [24,25]; and the benefits of a high FV consumption [26,27]. Furthermore, recent work of ours reported not only low knowledge of the details of the recommendations, but also a direct association between low knowledge of these details and low FV consumption [15]. These findings suggest that FV consumption would benefit from increasing knowledge of the details of FV recommendations.

Nutrition-related knowledge has previously been related to FV consumption [29-31] and is traditionally increased through educational campaigns and classes [1,31-33]. Educational campaigns, however, can be limited in scope, and classes can be limited in reach [32,33]. Furthermore, educational campaigns typically demonstrate success for improving very limited knowledge, whereas educational classes can achieve increases in knowledge and can be particularly valuable for teaching practical knowledge and for encouraging engagement, knowledge retention, and future use, but these are impractical for population-wide change [32,33].

This study sought to increase knowledge of the details of the UK FV recommendations both in a practical sense by providing details of the FV recommendations at the time of consumption to aid appropriate FV intakes, and by providing these details in an engaging, useful, and personally relevant manner, such that individuals would remember and benefit from those details also at a later time point [31-33]. To fulfill this aim, a mobile phone app was developed. Mobile phone apps can provide information to consumers at the time of food purchase and consumption, as well as outside of these times, and can offer an interactive platform encouraging practical use, personal relevance, and practical benefit to encourage information retention and future use. Of specific relevance to this study, an app was initially considered suitable for developing knowledge on FV recommendations because an app could allow users to input FV and receive immediate feedback on inclusion or not in the FV recommendations; allow users to input any amount of FV, regardless of contribution to an official portion, and receive immediate feedback on portion sizes; store and add inputted FV to provide a running total; relate this total to recommendations; incorporate the need for variety as part of the portion size and running total function; provide all information quickly, with minimal effort for the user; utilize attractive and colorful visual displays; and an app could be mobile and so could address concerns at the time of purchase and at the time of consumption, as well as outside of these times. The potential value of mobile phone apps is also aided by rapidly growing numbers of mobile phone users with penetration rates of 68.4% in North America and 64.7% in Western Europe and estimations of use by over a third of the world’s population [34].

Mobile phone apps for encouraging healthy eating are widely available, and some also focus specifically on encouraging FV intakes [35-40]. Although these existing FV apps largely focus on encouraging intakes and changing behavior [35-40], the focus of our study was to facilitate knowledge of FV consumption recommendations, such that this would result in increased adherence to recommendations and intakes. Previous work demonstrates particular confusions with FV recommendations and suggests that clarification of this knowledge may encourage FV consumption. Our aim was primarily to impart knowledge and facilitate retention and future use of that knowledge, such that FV intakes would benefit both at the time of app use and in the future without the need for ongoing app use.

### Objectives

This paper reports the development of a prototype app, the results of a subsequent pilot trial to test the app for improvements in FV recommendations knowledge and intake, and suggested improvements. First, the requirements for the app were defined from the literature and potential end users and prioritized using the Must have, Should have, Could have, Won’t have (MoSCoW) method [41]. Then, a prototype app was designed and developed using an agile approach based on Google’s Material Design Guidelines and best industrial practice [42]. The prototype app was then tested in a randomized controlled trial, where end users also provided qualitative feedback, and finally, an amended version of the app was developed. The app was developed specifically for a UK audience; thus, current UK FV recommendations were used. These recommendations specify the consumption of 5 80-g portions of different FV per day—the 5-a-day FV recommendations [43,44].

## Methods

### Stage 1: Defining and Prioritizing the App Requirements

#### Defining the App Requirements

App requirements were defined based on previous published research and engagement with potential end users. Previous published work by us and others has investigated the confusion and concerns of individuals regarding FV recommendations [15-20,23-28].

Further engagement with potential end users was also undertaken at 4 public engagement workshops in Bournemouth, United Kingdom, in July 2014 and July 2015. These workshops were undertaken as part of Bournemouth University’s Festival of Learning 2014 and 2015 and were entitled *The 5-a-day fruit and vegetable message* and marketed for the general public. The workshops detailed current FV recommendations for the United Kingdom, asked consumers for their knowledge and confusions, addressed these confusions, and provided advice for increasing intakes. Finally, participants were asked for the appropriateness of an app to help solve their confusions and encourage intakes. A total of 4 workshops were held at a number of different times in the day to allow attendance by a range of different individuals. Each workshop was run by the project PI (KMA) and either audio-recorded and transcribed or notes of all suggestions were taken at the time by an additional researcher. Each workshop followed the same format. All transcriptions and notes were subsequently analyzed using thematic analysis.

#### Prioritizing the App Requirements

Suggested requirements for the app from both the literature and the public engagement workshops were then discussed and prioritized by the principal researchers (KMA and NJ) using MoSCoW principles. The MoSCoW method [41] is a technique used in software development to prioritize the importance of the delivery of all identified requirements. Requirements are categorized as *must have*, *should have*, *could have*, and *won’t have*, based on importance, and then prioritized during the development process in this order. Requirements identified as *must have* are considered central to project success; those identified as *should have* are considered important, but not necessary; those identified as *could have* are considered desirable but not necessary; and those identified as *won’t have* are considered least important [41]. Consideration was also given to the UK FV recommendations. For example, the UK recommendations stipulate that 5 different FV must be consumed per day; thus, additional consumption of eaten FV would not contribute to the 5-a-day total, and that fruit juices/smoothies can contribute to total FV consumption but can only count as 1 portion regardless of variety and quantity consumed [43,44].

### Stage 2: Designing and Developing the App

A prototype app was developed to include all requirements identified as *must have* and *should have* and avoid requirements identified as *won’t have*. The app was developed for Android (Google) mobile phones following Google’s Material Design Guidelines and industrial best practices, with reference to the adapted technology acceptance model (TAM) [45-47]. The adapted TAM proposes that technology usage is positively predicted by *perceived usefulness* (“the degree to which a person believes that using a particular system would enhance his or her performance”) [45], *perceived ease of use* (“the degree to which a person believes that using a particular system would be free of effort”) [45], *perceived enjoyment* (“the extent to which the activity of using the [technology] is perceived to be enjoyable in its own right, apart from any performance consequences that may be anticipated”) [46], and *perceived visual attractiveness* (the degree to which a person believes that the [technology] is aesthetically pleasing to the eye) [47].

### Stage 3: Testing of the Prototype App

Evaluation of the app was undertaken using a randomized controlled pilot trial, where volunteers were randomized to receive or not receive the app for either 2 or 4 weeks, and FV knowledge, FV intakes, and FV behavior were assessed and compared at baseline and after either 1 and 2 weeks, or after 2 and 4 weeks.

#### Volunteers

Volunteers to test the app were recruited from the staff and students of Bournemouth University, United Kingdom, from November 2015 to March 2016, June 2016 to August 2016, and from November 2016 to March 2017. We aimed to recruit 100 volunteers in total—50 to test the app and 50 to act as controls. No earlier research was available to allow power calculations; thus, 50 volunteers were considered sufficient to gain feedback and assess potential impacts of the app, while ensuring the work would remain ethical should few impacts be found. Adult volunteers (aged 18 years and over) were required to own an Android mobile phone (as the app was only developed for Android platforms), and there were no other inclusion/exclusion criteria to maximize the generalizability of the study. Volunteers were recruited for a study to *test a novel mobile phone app for encouraging healthy behaviors*. Volunteers were thus aware at the study start, that they may or may not receive an app to test, but they were informed that the app may target one of a number of health behaviors, such as healthy eating, stress reduction, or exercising.

#### Intervention/Control

Volunteers were randomized to receive the app (intervention) or not receive the app (control). Randomization was undertaken on study entry by drawing lots (participants selected 1 of 2 colored dice from a bag), and recruitment stopped once 50 individuals had been randomized to test the app. All volunteers who received the app were asked to download the app onto their phones, to register with the app to set up a user profile, and to use the app as often as they wished for either a 2-week or a 4-week period. Duration of the test period for 2 or 4 weeks was undertaken to estimate effects following very short- and longer-term use. Various evidence suggests that apps can have limited effects on behavior because an initial high use typically fades [38,39]. Initial download and access to the app were undertaken in the presence of the researcher where possible to ensure correct download. No additional information on the FV recommendations or on FV intakes was provided as part of the study to either group. The only difference between the intervention and control group was the receipt of the app (intervention group) or not (control group). The app was tested for 2 weeks from November 2015 to March 2016 and from November 2016 to March 2017, and for 4 weeks from June 2016 to August 2016.

#### Outcomes

Awareness of the 5-a-day FV recommendations, FV knowledge, FV intakes, and FV behavior were assessed as outcomes. Awareness of the recommendations, FV knowledge, and self-reported FV intakes were assessed using a questionnaire previously developed by us [15]. The questionnaire consists of 2 questions on awareness of the 5-a-day message, 4 questions on knowledge of the details of the message (which foods are included, portion sizes, the need for variety, and reasons for consumption), and 2 questions on FV intake. Self-reported FV intake was also assessed using a validated Food Frequency Questionnaire (FFQ)—the Leeds Food and Nutrition Survey [48]. FV behavior was assessed using a behavioral measure of complementary drink choice. Demographic and lifestyle characteristics that have previously been associated with FV consumption and dietary knowledge [11,15-17,21,28] were also assessed as potential confounders. All volunteers (intervention and control) completed all outcome assessments in the same manner. To maximize the data collected in the study period, data were collected from those in the 2-week study at baseline, week 1, and week 2, and from those in the 4-week study at baseline, week 2, and week 4. The 2 self-report questionnaires used [15,48] are discussed briefly further and provided in Multimedia Appendix 1.

#### Awareness of the Recommendations

Awareness of the recommendations were assessed using 2 open-response questions: *Are you aware of the 5-a-day fruit and vegetable message?* and *What do you think it means?*

#### Fruit and Vegetable Knowledge

FV knowledge was assessed using 4 structured closed-response questions on (1) the FV that are included in the UK recommendations; (2) the portion sizes that are required for the recommendations; (3) the variety of FV that is required for the UK recommendations; and (4) the reasons for FV consumption. These questions include (1) a number of foods; (2) a number of different portions of FV; (3) a number of combinations of FV to be consumed in a day; and (4) a number of different health conditions, respectively, and respondents were asked to report (1) inclusion in the recommendations or not; (2) contribution to the recommendations based on portion sizes; (3) number of FV portions consumed in the day; and (4) the impact of FV on each health condition, respectively. For all questions, a correct response, based on current recommendations from the UK Government [44] is scored +1, an incorrect response is scored −1, and *don’t know/not sure* is scored 0.

#### Self-Reported Fruit and Vegetable Intake

FV intake was assessed using 1 single open-response question, 1 structured open-response question, and a validated FFQ [48]. The open-response question asked for estimated number of portions of FV consumed per day, to provide a measure of *Estimated FV*. The structured open-response question requested household amounts (eg, tablespoons) of all FV consumed at various time points (before breakfast, breakfast, morning, lunch, afternoon, evening meal, and evening) on a typical weekday and on a typical weekend day. This questionnaire was used to calculate portions of FV consumed per day, to provide a measure of *calculated FV*. The validated FFQ [48] requests frequency of consumption for 65 different foods using the response format *2 or more times a day*, *every day*, *3 to 5 times a week*, *1 to 2 times a week*, *1 to 3 times a month*, and *rarely/never*, which are subsequently scored *2*, *1*, *0.5*, *0.21*, *0.07*, and *0*, respectively, to provide a measure of frequency of consumption per day. The questionnaire was validated in adults at the time of development. A total of 10 questions on FV are provided, and responses to these 10 questions were then converted to consumption per day and summed, to give a measure of *FFQ FV*.

#### Fruit and Vegetable Behavior

FV intake was also assessed using a behavioral measure. Volunteers were offered a drink while completing all questionnaires and given the choice of tea, coffee, water, or fruit smoothie. The UK 5-a-day recommendations include fruit juice and fruit smoothies as FV [43,44]; thus, selections of the fruit smoothie were considered an FV choice, whereas all other drinks were considered a non-FV choice. No drink was also a permitted option.

#### Demographic and Lifestyle Characteristics

Demographic and lifestyle characteristics also assessed were gender, age, marital status, living status, number of years of education, smoking habits, alcoholic drinking habits, dietary supplement taking habits, and height and weight (to calculate body mass index).

#### App Feedback

Number of uses were requested from volunteers who received the app and downloaded from the app itself. Volunteers who received the app were also asked to give feedback on their experiences and offer suggestions for the app. This feedback was requested as part of the study debrief. Participants were free to offer as many or as few comments as they wished in a written or verbal form.

#### Additional Measures

To encourage a perception that the study was investigating the impacts of a number of apps for a variety of health behaviors, some additional measures, for example, questions on physical activity and stress, were also undertaken. These data were not analyzed.

#### Procedure

Volunteers undertook all outcome assessments at the Eating Behaviours Laboratory, Bournemouth University, United Kingdom. On each assessment occasion, volunteers completed all questionnaires using a Web-based platform (Qualtrics), were offered a drink, and had every opportunity to ask questions. One researcher randomized all volunteers and dealt with all queries, whereas another researcher oversaw all outcome assessments; thus, this researcher was blind to treatment (intervention/control).

The study was given ethical approval by the Research Ethics Committee of Bournemouth University before commencement and was registered as a clinical trial on ClinicalTrials.gov (NCT02779491). Methods were undertaken as detailed in the trial registration with the exception that a behavioral measure of FV intake was added to the study before commencement, and a measure of FV attitudes was cut. The original study proposal included a measure of attitudes toward FV, but these were decided against before the study start to reduce demand characteristics given the extensive FV knowledge questionnaire. All participants provided written informed consent before starting the study.

#### Analysis

Quantitative data were analyzed on an intention-to-treat basis, where missing data were completed using multiple imputation [49], based on gender, age, study period, and baseline measures. Demographic and lifestyle variables and all measures at baseline were first described and compared using 2-tailed *t* tests, on the basis of study duration and intervention/control grouping. To investigate impacts of the app with time, all FV knowledge and intake outcomes were analyzed using analysis of variance (ANOVA) for differences between baseline and week 2, and baseline and week 4. A covariate of study duration was also added to the ANOVA for the 2-week data, to accommodate differences between those studied for 2 weeks and those studied for 4 weeks. Thus, effects at week 2 were investigated using a 2 (intervention/control) × 2 (baseline/week 2) mixed analysis of covariance, and effects at week 4 were investigated using a 2 (intervention/control) × 2 (baseline/week 4) mixed ANOVA. Our behavioral measure of FV intake—choice of fruit drink or nonfruit drink was analyzed using chi-square tests. All data are reported as means and standard deviations. Significance was set at *P*<.05. Qualitative comments were analyzed using thematic analysis.

### Stage 4: Development of the Final App

Finally, the results and feedback from the users of the pilot randomized controlled trial were used to suggest amendments to create a final version of the app.

## Results

### Stage 1: Defining and Prioritizing the App Requirements

#### App Requirements

Previous published work reveals confusion around the foods that are included in the recommendations; the amount of FV required for a portion, particularly where large items, small items, and composite dishes do not always contribute complete portions; the number of portions needed per day; and the need for a variety of FV [15-20,23-28].

The 4 workshops were attended by 32 members of the population of Bournemouth. We did not measure any demographic variables, but individuals were noticeably of both genders, aged from 18 years to old age, and based on their questions or self-disclosures were students, mothers of young children, working professionals, and retired individuals.

These participants voiced similar confusions to those found in the literature and suggested that an app would potentially be appropriate to aid with these concerns. A total of 5 key themes emerged from analysis of the workshop discussions.

##### 1. Useful for Portion Sizes

Participants expressed particular difficulties over the differing portion sizes required for differing FV, and valued an idea that amount consumed could be entered into an app using household measures, for example, spoonfuls, and converted into portion sizes for them:

Oh yeah, that would be cool, so I can type in like 10 grapes, and it tells me, yeah, that’s one portion...or that’s only half a portion, or whatever...yeah, that would be handy.

If it could tell me my stew gives me two portions, when I have loads of veggies in it, just all in pieces,...then that would be handy.

##### 2. Useful Monitor

The app was considered likely to be useful for keeping track of FV consumption, particularly for small amounts of FV, for example, in composite dishes:

I like the idea that I might be getting 5 a day already but I just don’t know it...but to have a little machine to keep track of it in the day for me, and then I can check at the end, that would be helpful.

##### 3. Useful Target

The calculator function was also considered useful for telling users how close they were to a daily target:

If you could have some sort of bar to tell you how close you were to the 5 a day, that would be useful...you know, a man who gradually fills up, or something similar.

##### 4. Useful to Have It Mobile

Potential users also liked the idea that the app would be with them whenever they needed it; thus, they could use it in the evening to recap at the end of a day, but they could use it also, at point of purchase or point of consumption:

So you could use it in the shop or in the canteen and just try, you know, if I had the salad I would have 3 half portions, but if I have the hot meal and two veg [vegetable portions] instead of the chips, that would be two portions—that would be better. I would never think like that normally.

##### 5. Possible Negative Monitor

A few reservations were also expressed around the feedback that users may receive following their use of the app and the possibility that this may be negative:

I think it’s a neat idea, but I wouldn’t want anything telling me I was bad, or not eating well enough...I wouldn’t use it in that case—it needs to be nice to me!

#### Requirement Priorities

The priorities for the app based on MoSCoW principles are presented in Table 1.

**Table 1:** Must have, Should have, Could have, Won’t have (MoSCoW) requirements for the app.

Serial number		Requirement	
**Must have**			
	1		Allow users to input FV^a^ consumed at any time and using household amounts, for example, number of items and number of spoonfuls.
	2		Provide users with a list of all FV for selection, as opposed to requiring manual input.
	3		Categorize FV (eg, fruits, vegetables, and salad items) to avoid overly long lists of FV items for inputting.
	4		Allow users to input part items/units, where only part items have been consumed, for example, in composite dishes.
	5		Provide immediate feedback on inclusion or not of the FV in the UK 5-a-day recommendations.
	6		Calculate contribution to a portion for the UK 5-a-day recommendations based on amount consumed.
	7		Allow fractions of portions in these calculations, but do not allow multiple portions of the same FV in any one day.
	8		Provide immediate feedback on contribution of the portion to the UK 5-a-day recommendations.
	9		Sum contributions of portions to provide a running daily FV total.
	10		Relate this running daily total to the recommendations of 5 FV per day.
	11		Provide immediate feedback on the daily FV consumption per day.
	12		Require users to set up an account to allow FV to be tracked on a personal basis.
	13		Ensure users data are retained on their own device, to ensure data protection and privacy.
**Should have**			
	14		Provide FV items using colored picture icons as well as FV names.
	15		Display total daily FV consumed in a graphical manner allowing representation also of the target, for example, using a filled bar.
	16		Provide constructive feedback to highlight if the amount consumed is insufficient to amount to a whole portion, for example, *an additional spoonful of xxx would provide a full portion*.
**Could have**			
	17		Store daily running totals over time to allow users to view their history.
	18		Provide a signal when the 5-a-day target was met, for example, applause sound.
	19		Provide a reward when the 5-a-day target was met, for example, a token to be traded for material gain.
**Won’t have**			
	20		Provide instructive advice based on user inputs, for example, *you need to eat more of xxx*.
	21		Require users to input additional information, for example, time and place.
	22		Allow users to amend FV consumption in the past

^a^FV: fruit(s) and vegetable(s).

### Stage 2: Designing and Developing the App

The app was developed using an agile approach as described by Google’s Material Design Guidelines and industrial best practices [42]. A user journey map was first created to visualize the timeline of interactions with the potential app from the landing page. Wireframes of each app screen were then produced using Balsamiq. These wireframes focused on app screen layout and content structure and were organized to reflect the user journey map. These wireframes were then mapped to mock-ups showing the actual visual designs for each screen. An interactive prototype was created using InVision, and from this, an Android app was developed using native Android Studio. Primary researchers (KMA and NJ) were consulted at each step for feedback.

**Figure 1:**
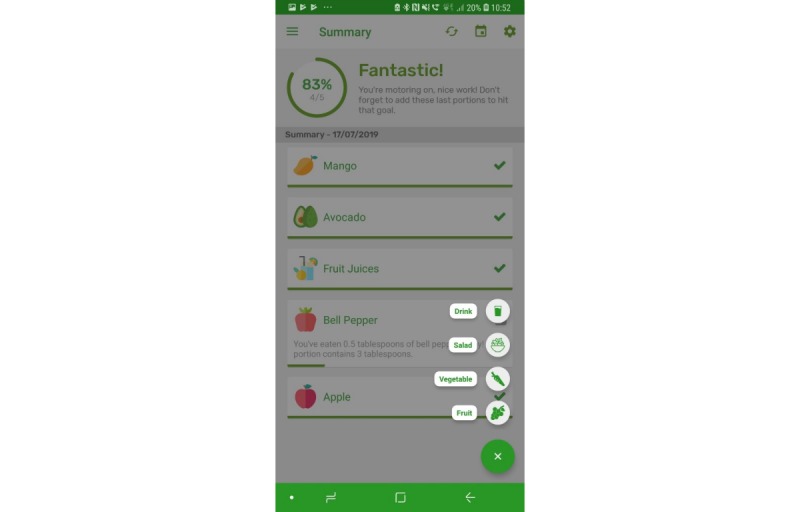
Screenshots of the app: fruit and vegetable categories.

**Figure 2:**
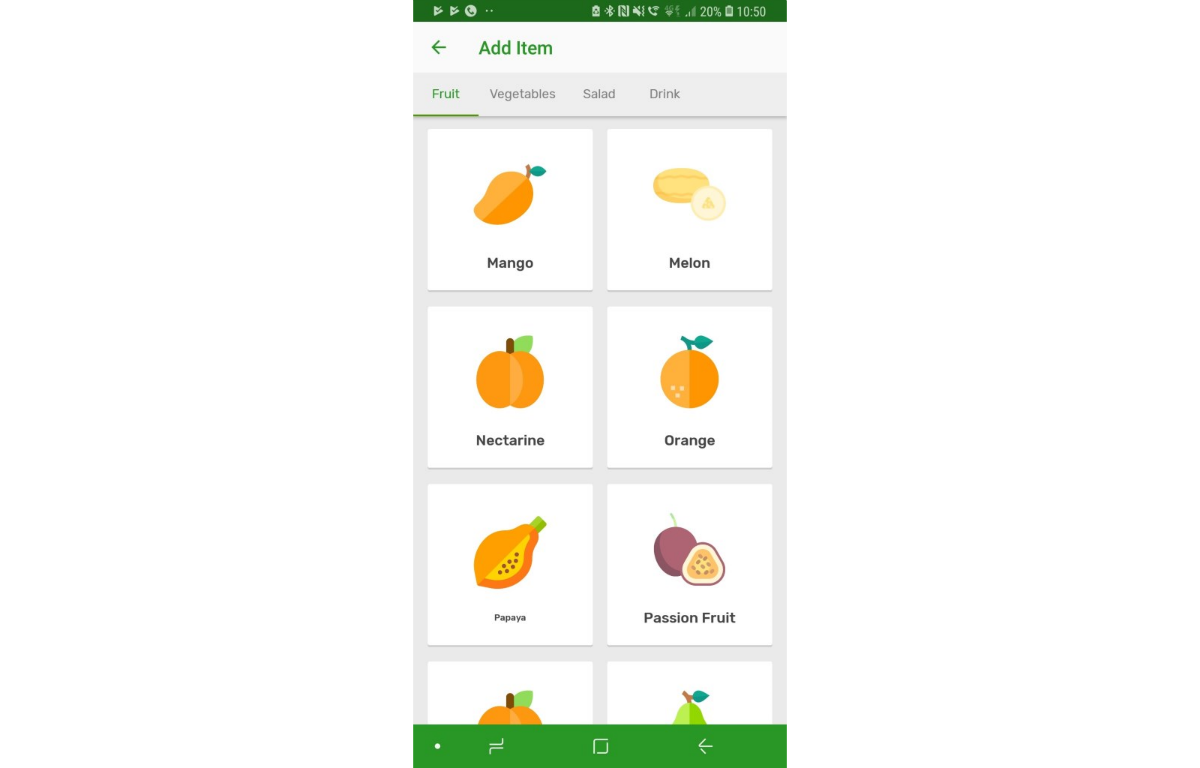
Screenshots of the app: fruit icons.

**Figure 3:**
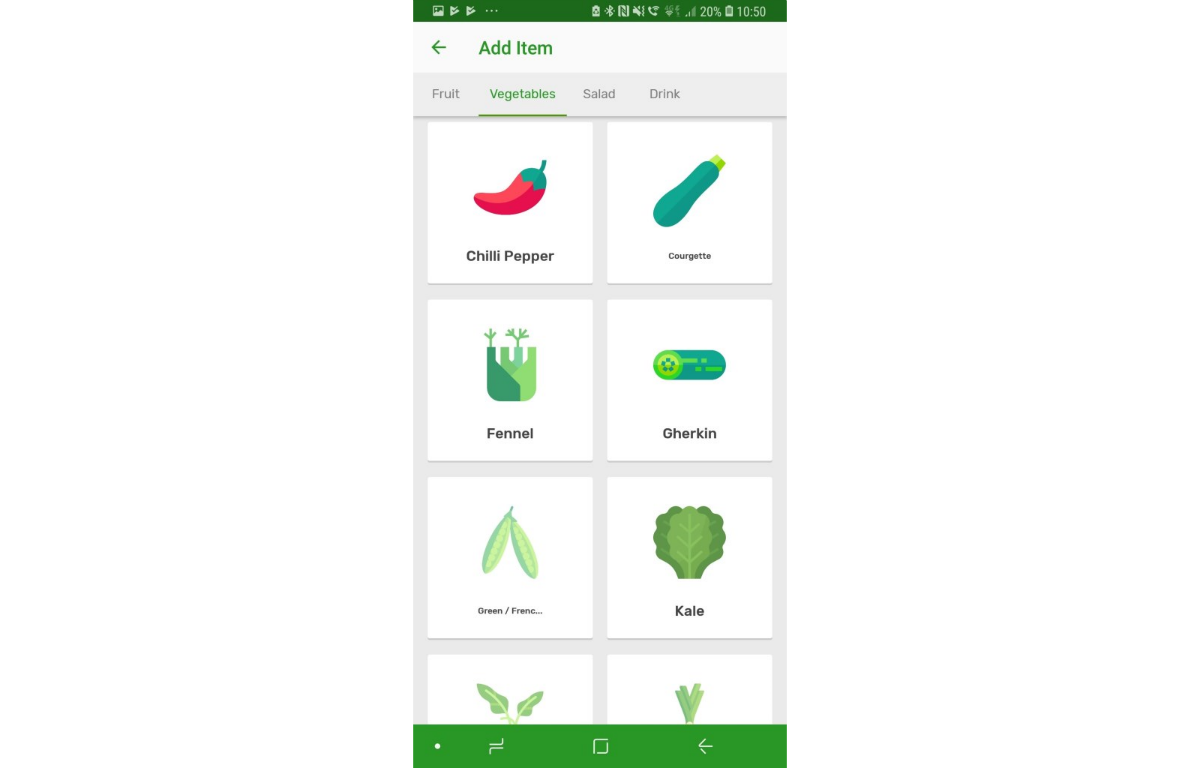
Screenshots of the app: vegetable icons.

**Figure 4:**
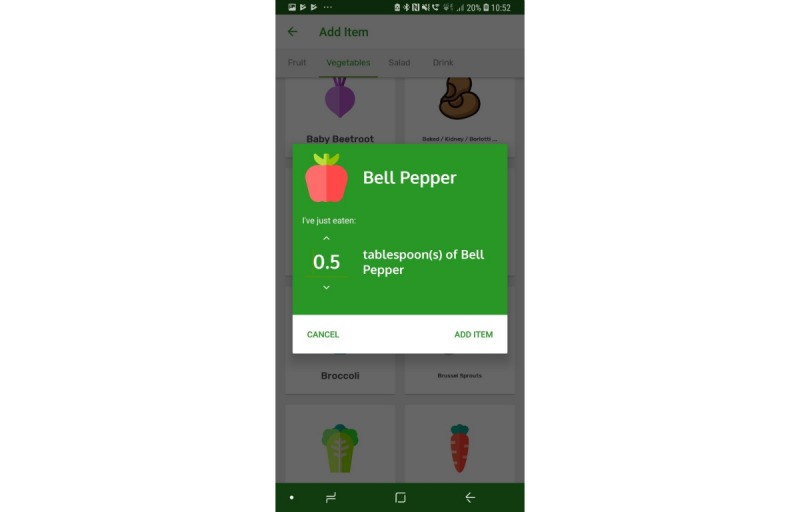
Screenshots of the app: fruit and vegetable selection.

**Figure 5:**
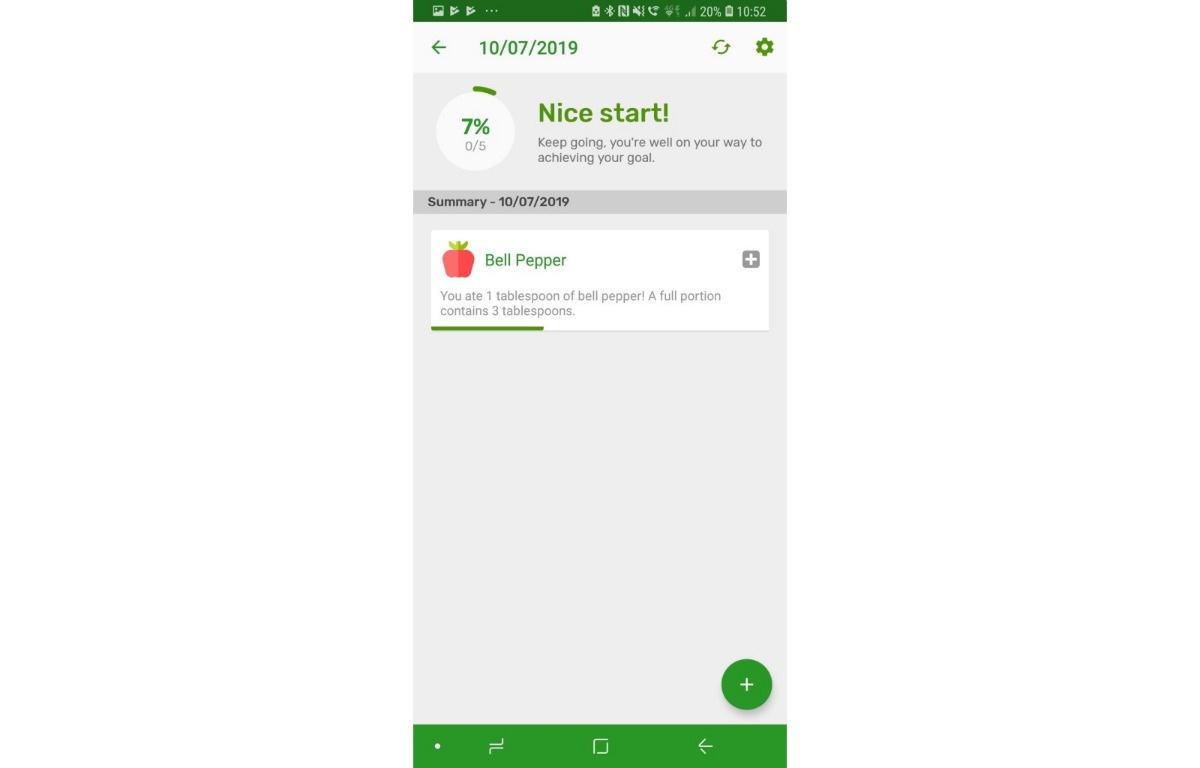
Screenshots of the app: Summary screen – low consumption.

**Figure 6:**
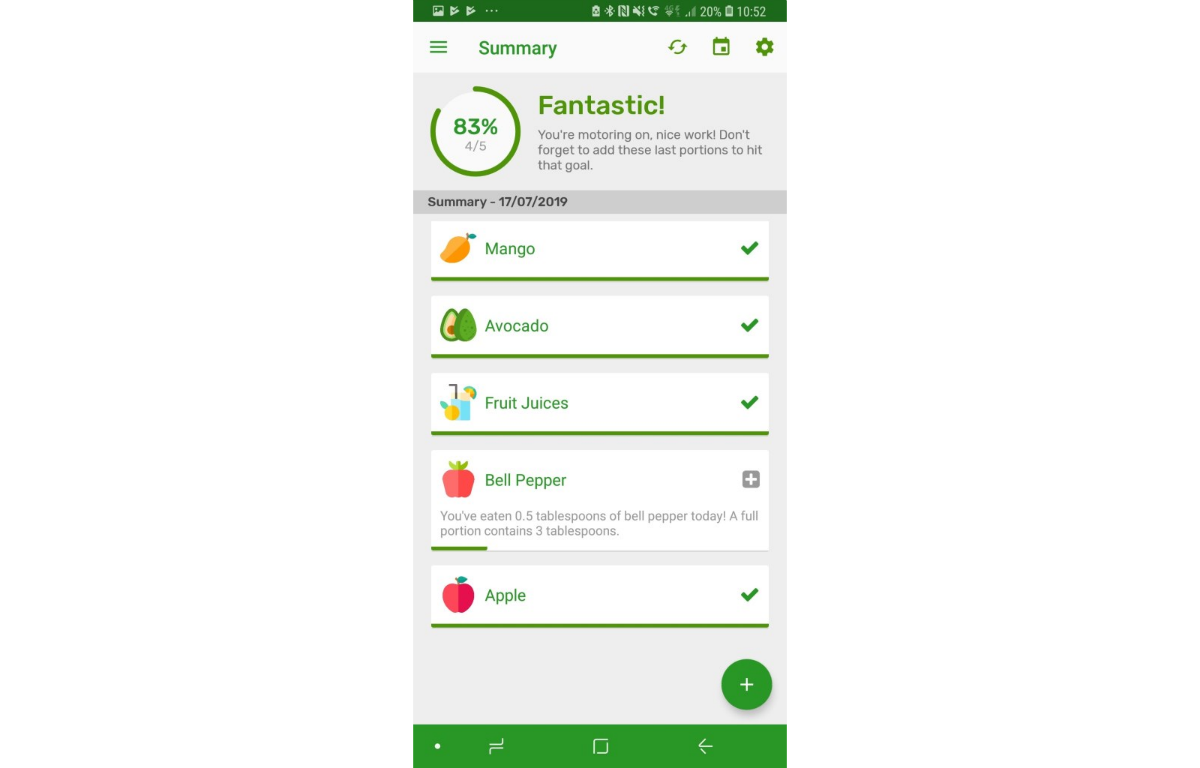
Screenshots of the app: Summary screen – high consumption.

The prototype app consisted of a series of screens allowing consumers to input and view their daily FV intake in comparison with the UK 5-a-day recommendations. All requirements identified as *must have* and *should have* were included with the exception that picture icons were not provided for some FV items (Table 1, requirement 14). Icons were not easily available for all FV items, and although desirable, icons for all FV items were considered not necessary at the prototype stage. Names were provided for all FV. All *won’t have* requirements were also avoided. Details of the app, per screen, are given in Table 2. Screenshots of screens 4, 5, 6, and 7 are given in Figures 1-6.

**Table 2:** Details of the prototype app.

Feature	Detail	Supported user actions	Requirements addressed
1. Welcome	The app name and app logo	Swipe to continue	—^a^
2. Registration	Request to login or register for an account	Provide a username to allow data to be tracked	12,13
3. Daily summary	Total FV^b^ inputted in the current day	Options to add (more) FV	11,15
4. Input categories	Lists of FV, categorized as *fruit*, *vegetables*, *salad,* and *drink*	Select relevant FV category	1-3
5. Input item	Individual FV items per category, displayed by name and icon (where available)	Select relevant FV item	1,2,4,5,14
6. Input amount	Arrows to select amount consumed, provided as items or spoons, as most commonly used	Select amount	1,4
7. Updated summary	Amount consumed provided in portions based on recommendations. Details of amount required for a full portion if <1 portion. Total FV inputted for the current day updated and displayed. Motivational or congratulatory message also displayed.	Options to add (more) FV	6-11,15,16

^a^No specified requirement addressed.

^b^FV: fruit(s) and vegetable(s).

### Stage 3: Initial Testing of the Prototype App

#### Volunteers

A total of 94 volunteers took part in the randomized controlled trial—50 who received and tested the app, and 44 who acted as controls. Of these, 32 volunteers received the app for 2 weeks, 27 volunteers acted as controls; 18 volunteers received the app for 4 weeks, 17 volunteers acted as controls. Demographic and lifestyle characteristics of all participants are given in Table 3. Volunteers who were studied for 2 weeks were more likely to be younger (*t*_92_=2.52; *P*=.02) and less educated (*t*_92_=4.08; *P*<.001), than those who were studied for 4 weeks, predominantly because volunteers were studied for a 2-week period when most of the volunteers were undergraduate students, and for 4 weeks when most of the volunteers were postgraduate students or university staff. No differences were found between the intervention and control groups in any demographic and lifestyle variable (largest *t*_57_=1.57; *P*=.12).

Adherence to the study was good. A total of 88 of 94 (94%) volunteers took part in all 3 test sessions, 1 volunteer undertook the first 2 sessions but failed to undertake the final session (control volunteer for 4 weeks), 3 volunteers undertook the first session but failed to undertake the second 2 sessions (1 volunteer received the app for 4 weeks, 1 volunteer was a control volunteer for 4 weeks, and 1 volunteer was a control volunteer for 2 weeks), and 2 volunteers undertook the first and third sessions but missed the second session (both volunteers received the app for 2 weeks). Reasons for dropout were not recorded.

**Table 3:** Demographic and lifestyle characteristics.

Characteristics		2-week study		4-week study	
		App (n=32)	Control (n=27)	App (n=18)	Control (n=17)
**Gender, n (%)**					
	Male	10 (31)	10 (37)	6 (33)	5 (29)
	Female	22 (69)	17 (63)	12 (67)	12 (71)
Age (years), mean (SD)		22.3 (7.7)	21.4 (5.3)	25.9 (7.9)	25.9 (7.9)
**Marital status, n (%)**					
	Married	0 (0)	0 (0)	1 (6)	2 (12)
	Not married	32 (100)	27 (100)	17 (94)	15 (88)
**Living, n (%)**					
	Alone	2 (6)	3 (11)	2 (11)	2 (12)
	With others	30 (94)	24 (89)	16 (89)	15 (88)
Education (years), mean (SD)		14.9 (1.7)	15.0 (1.5)	16.9 (2.8)	16.9 (2.8)
**Smoking status, n (%)**					
	Nonsmoker	27 (85)	21 (78)	16 (89)	13 (18)
	Light (0-2/day)	2 (6)	3 (11)	2 (11)	2 (12)
	Moderate (2-10/day)	3 (9)	2 (7)	0 (0)	1 (6)
	Heavy (10-20/day)	0 (0)	1 (4)	0 (0)	1 (6)
**Use of supplements, n (%)**					
	Never	18 (56)	14 (52)	5 (28)	8 (47)
	Occasionally	7 (22)	11 (41)	11 (61)	7 (41)
	Regularly	7 (22)	2 (7)	2 (11)	2 (12)
**Alcohol consumption, n (%)**					
	Never	4 (13)	2 (7)	6 (33)	3 (18)
	Light	20 (62)	19 (70)	7 (39)	9 (53)
	Moderate	7 (22)	9 (33)	4 (22)	4 (23)
	Heavy	1 (3)	0 (0)	1 (6)	1 (6)
Body mass index (kg/m^2^), mean (SD)		21.6 (8.4)	24.0 (5.5)	25.0 (4.4)	25.1 (4.3)
**Activity, n (%)**					
	None	1 (3)	1 (4)	0 (0)	0 (0)
	Standing all day	3 (9)	6 (22)	0 (0)	2 (12)
	Light	4 (13)	3 (11)	3 (17)	2 (12)
	Moderate	10 (31)	7 (26)	4 (22)	6 (35)
	Heavy	12 (38)	7 (26)	10 (55)	6 (35)
	Very heavy	2 (6)	3 (11)	1 (6)	1 (6)

#### Fruit and Vegetable Outcomes

Details of all FV outcomes are given in Table 4. Analyses of FV outcomes at baseline again revealed significant differences between volunteers studied for 2 weeks and those studied for 4 weeks in estimated FV consumption (*t*_92_=3.46; *P*<.001) and FFQ FV intakes (*t*_92_=2.49; *P*=.02). Volunteers studied for 4 weeks estimated and reported higher FV intakes. No differences were found in FV knowledge (largest *t*_92_=1.63*; P*=.11). No differences were found between the intervention and control groups at baseline (largest *t*_92_=1.10; *P*=.28).

**Table 4:** Mean (SD), fruit and vegetable knowledge scores, self-reported intake, and drink choice for all volunteers at baseline and at weeks 1, 2, and 4.

Outcome Variable		App									Control						
		Baseline (n=50)		1 week (n=32)		2 weeks (n=50)		4 weeks (n=18)		Baseline (n=44)		1 week (n=27)		2 weeks (n=44)		4 weeks (n=17)	
**FV^a^ knowledge, mean score (SD)**																	
	Foods (scored −35 to +35)		18.4 (6.8)		22.9 (6.4)		21.5 (8.1)		19.3 (7.9)		17.9 (7.1)		18.1 (8.8)		20.5 (8.3)		21.6 (6.3)
	Portion sizes (scored−27 to +27)		−6.4 (6.2)		−5.0 (8.0)		−4.4 (7.8)		−7.6 (9.5)		−7.8 (5.7)		−6.7 (5.7)		−6.4 (7.5)		−5.3 (7.4)
	Variety (scored −18 to +18)		−2.8 (5.7)		−0.4 (5.6)		0.1 (6.4)		−3.3 (6.3)		−2.6 (5.5)		−1.4 (6.0)		−1.1 (5.9)		−0.8 (6.9)
	Reasons (scored −25 to +25)		0.1 (5.7)		0.8 (5.7)		1.1 (4.8)		1.2 (5.6)		−0.1 (6.5)		−0.6 (5.3)		0.7 (5.2)		1.4 (5.9)
**FV intake, mean (SD)**																	
	FV estimated (FV portions/day),		3.3 (1.4)		2.9 (1.4)		3.4 (1.5)		4.1 (1.2)		3.2 (1.7)		3.1 (1.5)		3.3 (1.7)		3.6 (2.2)
	FV calculated (FV portions/day)		4.1 (1.4)		3.7 (1.5)		4.1 (1.6)		5.3 (1.6)		3.7 (2.0)		3.6 (2.2)		3.8 (1.8)		3.9 (1.6)
	FV FFQ^b^ (daily FV intake [portions])		3.5 (1.9)		2.6 (1.1)		3.2 (1.8)		3.5 (1.9)		3.3 (2.2)		2.3 (1.3)		2.9 (2.3)		3.9 (2.9)
**FV behavior, n (%)**																	
	Drink choice—Fruit smoothie		13 (21)		—^c^		23 (45)^d^		4 (25)		11 (36)		—		8 (16)^d^		5 (31)
	Drink choice—Other drink		22 (18)		—		8 (16)^d^		5 (31)		15 (25)		—		12 (23)^d^		2 (13)

^a^FV: fruit(s) and vegetable(s).

^b^FFQ: Food Frequency Questionnaire.

^c^Data not collected at this timepoint.

^d^Significant differences between the app and control groups (χ^2^_1_=6.0; *P*=.02).

#### Fruit and Vegetable Awareness

All volunteers with the exception of 2 volunteers in the 4-week study (1 who received the app and 1 who was a control) were aware of the 5-a-day FV recommendations at baseline, and at sessions 2 and 3, all volunteers were aware of the recommendations.

#### Fruit and Vegetable Knowledge

Significant increases by week 2 were found for the questions on foods included in the recommendations (*F*_1,91_=5.11; *P*=.03) and portion sizes (*F*_1,91_=5.69; *P*=.02), and by week 4 for all FV knowledge questions (smallest *F*_1,33_=4.65; *P*=.04). No differences were found between the intervention and control groups with time (largest *F*_1,33_=1.03; *P*=.32).

#### Fruit and Vegetable Intake

No differences were found between the intervention and control groups over time (largest *F*_1,91_=0.44; *P*=.51). Significant differences based on study duration were retained in estimated FV and FV intakes assessed by FFQ (smallest *F*_1,91_=7.83; *P*=.01). Correlations among all 3 FV intake measures also demonstrated comparability (smallest r=0.41; *P*<.001). No effects of time were found (largest *F*_1,91_=1.73; *P*=.19).

#### Fruit and Vegetable Behavior

No significant differences between groups were found at baseline (χ^2^_1_=0.17; *P*=.68). By week 2, significantly more fruit smoothies were chosen by those in the intervention group compared with controls (χ^2^_1_=5.96; *P*=.02), but no effects were found at week 4 (χ^2^_1_=1.17; *P*=.28).

#### App Usage

Self-reported usage of the app was high—most participants reported using the app on most days or every other day. Recorded use of the app also suggested almost daily usage or usage every other day. Following initial access, volunteers in the 2-week study used the app for a mean of 11.4 (SD 7.2) times, ranging from 0 to 27 times, and volunteers in the 4-week study used the app for a mean of 13.7 (SD 9.2) times, ranging from 2 to 34 times. App usage was greater in the earlier part of each test period. On the majority of days on which it was used, the app was used only once. In total, the app was used once a day on 63.6% (232/365) days on which the app was used in the 2-week study and on 65.6% (162/247) days on which the app was used in the 4-week study; twice a day on 31.8% (116/365) days and 22.3% (55/247) days, respectively; 3 times a day on 3.8% (14/365 total) days and 9.7% (24/247) days, respectively; 4 times a day on 0.8% (3/365) days and 1.0% (3/247) days, respectively; and 5 times a day on 1.0% (3/247) days on which the app was used in the 4-week study.

#### App Feedback

Qualitative feedback on the app was positive—almost all volunteers reported liking the app although many also reported room for improvements. Suggested improvements included an option to add FV for the previous day because these were possibly simply forgotten; an option for changing the goal from 5 a day to more than this if individuals preferred to aim higher; a need for missing FV to be added, or an option to give feedback that FV were missing so that these could be added; a daily notification or option to add these to remind users to interact with the app; and tips or suggestions for how to increase FV consumption.

The majority of volunteers also reported that the app was useful. Almost all volunteers reported that the app was useful for keeping a record of their consumption and for making them aware of limited consumption:

Made me conscious of what I was eating.

Good to have a record of how much of 5-a-day was eaten and also to know when you’re short.

Volunteers also reported increased FV intakes through a wish to engage with the app:

I think it made me want to eat more fruit and veg because I had to write it down.

...and adhere more fully with recommendations:

I think it was useful in terms of realizing that I don’t eat enough fruit and veg, as it has made me think about it more.

I would eat more at dinner if I noticed I had not eaten enough that day.

A limited number of volunteers also felt that the app was unnecessary:

The app was useful, but I personally don’t need an app to ensure that I get my 5 a day.

For some, it did not help them:

Did not help as I plan meals the week before.

### Stage 4: Development of the Final App

On the basis of the outcomes and feedback from the pilot test, a second version of the app is under development. Amendments that have so far been completed are to include picture icons for all FV items included in the app; to ensure more FV are included on the app; to allow users to return to previous days to add additional items where desired; and to allow users to change the target FV to more than 5 if desired (the default setting is for a target of 5 FV per day). A *history* option allows users to return to a previous day to add additional items. An ability to return to previous days was initially avoided in the prototype app to discourage users from adding false information as a result of faulty recollections. Considering that the app is primarily for the benefit of the user and that false information can be added to the app at any time, requests for access to previous days has been granted and may be beneficial for some users. The option to change the intake goal is presented to users at registration and can be amended as desired as part of the user profile settings. The additional screens for the final app are given in Table 5; all screens for the prototype app also remain.

**Table 5:** Additional screens of the final app.

Feature	Detail	Supported user actions	Requirement addressed
8. Personal preferences	Options for *History* to allow inputs for previous days; *Reports* to provide an overview for the week; *Refresh* to request updates; *Settings* to update goal targets and add notifications.	Select options or return to Summary (feature 3)	—^a^
9. History	Calendar display	Select date, input FV^b^ as for the current day	Historical input permitted
10. Reports	Overview of FV intake for the previous week/month (not yet enabled)	—	—
11. Refresh	Refreshes and updates total	Enabled	—
12. Settings	Options for *Help* to feedback to the developer; *User* to access details of the user and amend intake goal; *Notifications* to set alarms; *Devices* and *App*.	Select options or return to Summary (feature 3)	—
13. Help	Abilities to contact the development team (not yet enabled)	—	—
14. User	User details and user setting displayed	Option to amend intake goal	Goal amendment permitted
15. Notifications	Abilities to set up notifications (not yet enabled)	—	—
16. Devices	Device details provided	—	—
17. App	App version details provided	—	—

^a^No specified user action or requirement addressed.

^b^FV: fruit(s) and vegetable(s).

Amendments that are still under development will allow users to reduce or delete an FV item once this has been logged (this is currently not possible); allow users access to an overview of FV consumed over the previous week or month; allow users to set up notifications; and allow users to give feedback directly to the development team. Consumption totals for previous days can currently be viewed individually, but a historical overview may also be helpful. An interactive notification is intended to demonstrate to users the further consumption required on any one day to meet the recommendations. The default setup will be for no notifications to avoid negative reactions to the app, but notification setup will also be easy if desired. Other suggestions from app users to include tips and suggestions to increase FV are not currently planned to retain the focus and simplicity of the app.

The final app is now available for download for Android mobile phones at no cost from the Google Play Store under the name of SMART 5-A-DAY. Development continues, and updated versions of the app will be released as new features are added.

## Discussion

### Principal Findings

A novel mobile phone app was conceived to increase knowledge of the details of the UK FV recommendations both in a practical sense by providing details of the FV recommendations at the time of consumption, and by providing these details in an engaging, useful, and personally relevant manner, such that individuals would remember and benefit from those details also at a later time point. A prototype app was developed and tested by 50 users as part of a randomized controlled pilot trial, for either 2 or 4 weeks. FV assessments and positive qualitative comments suggested positive impacts of the app, but reported effect sizes were small. Additional features were suggested, and a final version of the app is currently under development.

The early development work confirmed low knowledge of the details of the 5-a-day FV recommendations in consumers, as found in the published literature [15-20,22,23], and reinforced the researchers’ suggestions on the suitability of an app for providing increased FV knowledge. App development was then possible as required, to result in a fully functioning interactive mobile phone app. The results of the randomized controlled pilot trial demonstrate limited impacts of the app on the questionnaire measures of FV knowledge and FV intakes, although an impact on FV behavior was found, and qualitative feedback suggested benefits. Improvements in FV knowledge were found across the study (regardless of app receipt) presumably as a result of inclusion in a study on healthy eating and the repeated assessment of FV knowledge and FV intakes, so increased awareness of these issues. The limited findings specific to those who received the app suggest that benefits of the app are small, particularly in addition to the benefits of taking part in the study, although increased FV knowledge in all study volunteers regardless of app/no app provision may have masked impacts of app use.

An impact on FV behavior was found. Given a choice of a range of available drinks, use of the app for 2 weeks resulted directly in increased FV selection and consumption. Behavioral outcomes are important, as it is only behavior that will impact on health [31-33], and we have previously suggested that spontaneous behavioral outcomes, such as those found here may be particularly valuable in an environment of plenty [50]. Small spontaneous changes in behavior such as this may also remain largely unnoticed by individuals themselves and so may go largely unreported in self-reported measures such as those also used and often included in studies such as these [51-53].

The qualitative feedback also suggested potential changes in intake, but again that these changes may be small and may go uncaptured by traditional dietary assessment methods [52]. Furthermore, the qualitative feedback goes on to suggest that these small changes may have occurred more as a result of volunteers becoming more aware of their intakes than previously and becoming particularly aware of low intakes. Awareness of a need for change has previously been suggested as an important step toward behavior change [33,34]. The qualitative feedback also fails to suggest impacts on FV knowledge, and although the app was intended to increase knowledge, it is well recognized that recording food intake can alert consumers to eating patterns, particularly some eating patterns that are not easily recognized over a whole day, and that this realization can change behavior [51,54-56]. The importance of increasing awareness of low intakes was not anticipated, but this finding suggests an added benefit from the app.

The qualitative comments were largely positive. Negative comments centered solely around a lack of personal interest or relevance because these users were already high FV consumers. High FV consumers are not the target audience for the app.

Additional findings from the initial test also related to app usage. Around 65% of those who received the app used it initially; these figures dropped throughout the test period, and the majority of users used the app once per day. These data are comparable with those found in studies of similar apps [38,39]. Our app was intended for use as often as volunteers wished—possibly once a day for record keeping or more often to acquire knowledge or encourage good adherence. The pattern of use suggests our testers were using the app more to track intake than to gain knowledge. These findings suggest that for maximum benefit from the app, it may be useful to market the app specifically for gaining knowledge of the recommendations and for adherence to these. This would also help distinguish our app from other apps that are intended primarily for tracking and record keeping [35-39]. Increased usage at the start of the usage period is commonly found in app testing studies, and the reduced subsequent usage is frequently cited as suggestive of poor engagement. Many users also suggested an additional reminder to aid interaction with the app, or requested an ability to return to a previous day to input forgotten items. These findings suggest that motivation to use the app was quite low among our testers, but our trial was not advertised as a study on FV consumption or healthy eating (to avoid demand characteristics); thus, our testers are likely to have been less motivated than those who would be more likely to use an app on 5-a-day FV recommendations of their own volition. Importantly, furthermore, on the basis of the qualitative comments, we do not consider this reduced usage to demonstrate poor functionality of the app. The app was intended mainly to encourage users to understand and learn the FV recommendations; thus, extended use should not be necessary.

The work conducted here further demonstrates the value of the early consultative work and the randomized controlled pilot test. Positive responses to the app overall demonstrate the value of the early research and the initial consultation exercises with potential end users. The increases in FV knowledge and intakes in all trial volunteers demonstrate the value of a randomized controlled trial for testing the app. Not all apps are tested for impacts on behavior before release, and many that are tested are done so without also involving a control group. Consideration in our study of only the 50 app testers would have suggested considerable increases in FV knowledge and intakes as a result of the app, whereas the inclusion of the control group demonstrates these impacts to probably result more from study inclusion or FV questionnaire completion.

Our randomized controlled trial was limited through the repeated assessment of FV outcomes and the repeated use of self-reported measures. These types of measures have previously been demonstrated as accurate [48,51-53], but very brief measures may have been insensitive to small changes. Our trial was also limited through the inclusion of testers who were not our intended target group. The app is intended for those who wish to improve their knowledge of the FV recommendations, probably to aid FV intakes. To avoid demand characteristics in our trial, we asked only for those who wished to try a new health-orientated app, and some of these individuals may have been unmotivated, unwilling or unable to improve FV intakes. By comparison, our testers were unsure of the apps being tested in the study, thus were unclear that FV was the focus for all users, and responses to the FV questionnaire in our volunteers did confirm low knowledge of the 5-a-day FV recommendations among the population [15-20,22,23]. Impacts based on age and education have also been found previously [11,22,23]. We could also have measured usability of the app using more formal measures, such as the System Usability Scale [57]. Considering the more comprehensive measures of app usage and app benefit in terms of knowledge and intakes in our trial, we did not collect these usability measures, but information from these measures may have allowed comparison with other apps or technological devices [57].

The positive responses and potential for changes in behavior have resulted in continued development of the app to result in an amended version. This version includes clear details of an FV consumption goal, allows users to input FV consumed and provides detailed and graphical information on how this consumption relates to the FV recommendations, provides clear personalized feedback on distance to the goal, and allows users to change their target FV goal to a goal of their choice as they wish. Our final app thus includes 3 key aspects of behavior that have previously been suggested to lead to successful behavior change, particularly for dietary behaviors, alongside increased knowledge: self-monitoring, goal-setting, and feedback in relation to goal attainment [54-56]. Other apps and interventions aiming to improve FV intakes and dietary quality also use similar behavior change techniques [35-40], and self-monitoring and goal-setting have previously been suggested as particularly important techniques by professionals [54-56] and by consumers [35,39]. It is interesting that although our app initially aimed to increase only knowledge, functions as a result of user feedback now also include established behavior change techniques.

The increased FV behavior and qualitative reports suggest that the app has potential to benefit FV intakes and health, although changes may be small. Small changes on a population-wide level, however, will have significant impacts. Increased benefit is also likely from the added features, from highlighting the knowledge component, from tests of the app in our target audience, and from the additional features still under development.

Our amended app now also needs testing. Further testing will not only demonstrate the improved value of the app but may also demonstrate the aspects of the app of particular benefit, given our inclusion also of behavior change techniques and knowledge and the reported value of these [54-56]. Additional functionality also allows direct linkage with additional software allowing direct access to questionnaires or other research materials.

Further development of the app may also be of value. Notably, our initial discussions with potential end users suggested an interest in both immediate and longer-term rewards for reaching a target goal. Repeated work demonstrates a value for rewards for encouraging healthy food consumption, including FV consumption [58], and rewards have previously formed an integral part of many successful dietary change interventions [36,55,59]. Other studies also suggest only limited benefit from apps for behavior change and have suggested a need for strategies to ensure continued use [36,40]. One of the advantages of our app was an intention that users would gain knowledge through the app; thus, extended use should not be required. Many apps related to social activities, such as eating, also include a *share* option to allow others to view the inputs of others or allow comparisons between users or with an established norm. Feedback options for others to comment on FV inputs, through *likes*, may also facilitate motivation, and so facilitate engagement with and action based on the app. Offline and different versions of the app may also be desirable, for example, through the use of different formats, different controls, or different setups, possibly for different population groups. Adolescents and young adults are groups with low FV consumption that may be particularly inclined toward digital interventions [39]. Socially deprived consumers may also benefit from specific aspects of the app, and investigation in different cultures (using local recommendations) would be of interest. Further work discriminating between those who use and do not use the app, and between those who find and do not find the app useful, would be very valuable.

### Conclusions

In conclusion, this study details the development and early test results of a novel interactive mobile phone app for improving knowledge and implementation of the UK 5-a-day FV recommendations. SMART 5-A-DAY was developed following assessment of the existing literature and consultation with potential end users, and then tested in a randomized controlled pilot trial. The trial demonstrated increased FV behavior 2 weeks after app receipt compared with control, and resulted in positive feedback, although resultant changes in FV knowledge and intakes were small. Suggestions for amendments were also made. Development of the app is ongoing, and further testing is required.

## Appendix

Multimedia Appendix 1 FV Knowledge Questionnaire (Appleton et al, 2017); Leeds Food and Nutrition Survey (Margetts et al, 1989).

Multimedia Appendix 2 CONSORT‐EHEALTH checklist (V 1.6.1).

## Abbreviations

ANOVA: analysis of variance
FFQ: food frequency questionnaire
FV: fruit(s) and vegetable(s)
MoSCoW: Must have, Should have, Could have, Won’t have
TAM: technology acceptance model
