# Supplementary material for: An Interactive Mobile Phone App (SMART 5-A-DAY) for Increasing Knowledge of and Adherence to Fruit and Vegetable Recommendations: Development and Pilot Randomized Controlled Trial
Source: JMIR Mhealth Uhealth. 2019 Nov 20;7(11):e14380. doi: 10.2196/14380 (PMC6893570; doi:10.2196/14380)
Supplement: Multimedia Appendix 1 [file mhealth_v7i11e14380_app1.docx]

Supplementary Materials:

FV Knowledge Questionnaire (Appleton et al, 2017); Leeds Food and Nutrition Survey (Margetts et al, 1989)

**EATING HABITS AND LIFESTYLES**

To answer the following questions, please think about the LAST MONTH, and list all fruits and vegetables you consume on a typical WEEKDAY.
Please include amounts, using items, tablespoons, cupfuls, whatever is most appropriate.
e.g. Weekday breakfast - 1 banana, 1 tablespoon of raisins, 1 glass of pure fruit juice

Please include fresh fruits and vegetables, pure fruit juice, tinned fruit and vegetables, frozen fruits and vegetables and dried fruits and vegetables.

**1. Weekday breakfast - Fruits and vegetables consumed, including amounts**

**2. Weekday midmorning -**

**3. Weekday lunch -**

**4. Weekday midafternoon -**

**5. Weekday evening meal -**

**6. Weekday evening -**

To answer the following questions, please think about the LAST MONTH and list all fruits and vegetables you consume on a typical WEEKEND day.
Please include amounts, using items, tablespoons, cupfuls, whatever is most appropriate.
Please include fresh fruits and vegetables, pure fruit juice, tinned fruit and vegetables, frozen fruits and vegetables and dried fruits and vegetables.

**7. Weekend day breakfast - Fruits and vegetables consumed, including amounts**

**8. Weekend day midmorning -**

**9. Weekend day lunch -**

**10. Weekend day afternoon -**

**11. Weekend day evening meal -**

**12. Weekend day evening -**

**13. On average, how many portions of fruit and**

**vegetables do you currently consume per day?**

**14. Are you aware of the 5-a-day message about eating fruits and vegetables?**

| □ yes | □ no – please skip to question 20 |
| --- | --- |

**15. What do you think the 5-a-day fruit and vegetable message means?**

**16. Following are a list of commonly consumed foods. Let us know whether you think each counts as a fruit or vegetable according to the 5-a-day message. For each item, answer: yes - this counts, no - this doesn't count, or don't know / not sure.**

|  | yes | no | not sure |
| --- | --- | --- | --- |
| apple |  |  |  |
| baked beans |  |  |  |
| broccoli |  |  |  |
| cauliflower cheese |  |  |  |
| cherries |  |  |  |
| chickpeas |  |  |  |
| chips |  |  |  |
| chocolate |  |  |  |
| coffee |  |  |  |
| crisps |  |  |  |
| dried apricots |  |  |  |
| fried onions |  |  |  |
| green beans |  |  |  |
| grilled tomato |  |  |  |
| jacket potatoes |  |  |  |
| lentils |  |  |  |
| melon |  |  |  |
| minced meat bolognaise sauce (meat, onions, tomato) |  |  |  |
| mushrooms |  |  |  |
| orange |  |  |  |
| plum |  |  |  |
| potatoes |  |  |  |
| pure fruit juice |  |  |  |
| raisins |  |  |  |
| red bell pepper |  |  |  |
| stew (meat, onions, root vegetables, potatoes) |  |  |  |
| strawberry jam |  |  |  |
| sultanas |  |  |  |
| sweetcorn |  |  |  |
| tinned tomatoes |  |  |  |
| tinned pineapple |  |  |  |
| tomato soup |  |  |  |
| vegetable lasagne |  |  |  |
| vegetarian pizza (onion, peppers, mushroom) |  |  |  |
| wine |  |  |  |

**17. Following are a list of commonly consumed foods. For each food, let us know how many portions of fruit and vegetables would be provided, according to the 5-a-day message. Just give us number of portions, and you can have part portions too.**

|  | 0 | 1/3 | 1/2 | 1 | 2 | not sure |
| --- | --- | --- | --- | --- | --- | --- |
| 1 apple |  |  |  |  |  |  |
| 1/2 banana |  |  |  |  |  |  |
| 1 heaped tablespoon of baked beans |  |  |  |  |  |  |
| 3 heaped tablespoons of carrots |  |  |  |  |  |  |
| 1 portion of cauliflower cheese (3-4 heaped tablespoons) |  |  |  |  |  |  |
| 7 cherries |  |  |  |  |  |  |
| 6 dried apricots |  |  |  |  |  |  |
| 1 heaped tablespoon of fried onions |  |  |  |  |  |  |
| 1 glass of pure fruit juice |  |  |  |  |  |  |
| 6 heaped tablespoons of fruit salad |  |  |  |  |  |  |
| 1 heaped tablespoon of green beans |  |  |  |  |  |  |
| 1 grilled half tomato |  |  |  |  |  |  |
| 1 portion of minced meat bolognaise sauce (3-4 heaped tablespoons) |  |  |  |  |  |  |
| 1/8 of a melon |  |  |  |  |  |  |
| 14 button mushrooms |  |  |  |  |  |  |
| 1 medium orange |  |  |  |  |  |  |
| 1 heaped tablespoon of peas |  |  |  |  |  |  |
| 1 red bell pepper |  |  |  |  |  |  |
| 2 slices of tinned pineapple |  |  |  |  |  |  |
| 1 plum |  |  |  |  |  |  |
| 1 heaped tablespoon of raisins |  |  |  |  |  |  |
| 1 portion of stew (3-4 heaped tablespoons) |  |  |  |  |  |  |
| 1 heaped tablespoon of sultanas |  |  |  |  |  |  |
| 3 heaped tablespoons of sweetcorn |  |  |  |  |  |  |
| 1 bowl (1/2tin) of tomato soup |  |  |  |  |  |  |
| 1 portion of vegetable lasagne (3-4 heaped tablespoons) |  |  |  |  |  |  |
| 1 portion of vegetarian pizza (half a 12 inch pizza) |  |  |  |  |  |  |

**18. Following are a list of commonly consumed foods, all consumed on the same day. For each day, let us know how many portions of fruit and vegetables would be provided that day, according to the 5-a-day message.**

|  | 1 | 2 | 3 | 4 | not sure |
| --- | --- | --- | --- | --- | --- |
| 1 apple, 1 pear |  |  |  |  |  |
| 1 apple, 1 banana and 1 glass of pure fruit juice |  |  |  |  |  |
| 1 apple, 3 tablespoons of green beans and 3 tablespoons of sweetcorn |  |  |  |  |  |
| 1 glass of pure fruit juice, 1 banana, 2 tablespoons of baked beans, 1 tablespoon of tinned tomatoes |  |  |  |  |  |
|  | 1 | 2 | 3 | 4 | not sure |
| 1 glass of pure fruit juice, 1 grilled tomato, 1 tablespoon of fried mushrooms, 2 tablespoons of baked beans |  |  |  |  |  |
| 1 apple, 1 pear, 2 glasses of pure fruit juice |  |  |  |  |  |
| 2 apples, 2 bananas |  |  |  |  |  |
| 3 tablespoons of baked beans, 3 tablespoons of lentils, 3 tablespoons of cooked tomatoes, 1 onion |  |  |  |  |  |
| 2 glasses of pure fruit juice, 2 tablespoons of sweetcorn, 1 tablespoon of green beans |  |  |  |  |  |
| 3 bananas |  |  |  |  |  |
| 1 portion of vegetable lasagne, 1 apple |  |  |  |  |  |
| 1 portion of vegetable lasagne, 2 tablespoons of chips, 1 glass of pure fruit juice |  |  |  |  |  |
| 1 portion of vegetable lasagne, 3 tablespoons of mixed vegetables |  |  |  |  |  |
| 1 glass of orange juice, 1 glass of apple juice, 1 banana |  |  |  |  |  |
| 2 tablespoons of baked beans, 2 tablespoons of sweetcorn, 2 tablespoons of cooked tomatoes, 1 onion |  |  |  |  |  |
| 2 tablespoons of cooked tomatoes, 1 tablespoon of baked beans, 1 onion, 1 carrot |  |  |  |  |  |
| 1 plum, 1 apricot, 1 banana |  |  |  |  |  |
| 2 bananas, 6 tablespoons of tinned peaches |  |  |  |  |  |

**19. Following are a list of common health conditions. Please let us know if you think each condition is helped by eating fruit and vegetables. For each condition, answer: yes definitely, this is aided by eating fruit and vegetables and plenty of evidence shows this; yes possibly, this may be aided by eating fruit and vegetables, but only a little evidence shows this; no, this is not aided by eating fruit and vegetables; or don't know / not sure**

|  | Yes definitely | Yes possibly | no | Not sure |
| --- | --- | --- | --- | --- |
| Acne |  |  |  |  |
| Alzheimers disease |  |  |  |  |
| Anxiety |  |  |  |  |
| Arthritis |  |  |  |  |
| Asthma |  |  |  |  |
| Body weight |  |  |  |  |
| some types of Cancer, such as bowel cancer |  |  |  |  |
| Cardiovascular disease |  |  |  |  |
| Common colds |  |  |  |  |
| Dementia |  |  |  |  |
| Depression |  |  |  |  |
| Type II Diabetes |  |  |  |  |
| Ezcema |  |  |  |  |
| Hypertension |  |  |  |  |

|  | Yes definitely | Yes possibly | no | Not sure |
| --- | --- | --- | --- | --- |
| Immune function |  |  |  |  |
| Influenza |  |  |  |  |
| Measles |  |  |  |  |
| Obesity |  |  |  |  |
| Osteoporosis |  |  |  |  |
| Rubella |  |  |  |  |
| Stress |  |  |  |  |
| Stroke |  |  |  |  |
| Sunburn |  |  |  |  |
| Tuberculosis |  |  |  |  |
| Typhoid |  |  |  |  |

**20. Date of completion:**

|  Jan | Feb | Mar |   Apr | May | June | July | Aug | Sept |   Oct | Nov | Dec |
| --- | --- | --- | --- | --- | --- | --- | --- | --- | --- | --- | --- |

**21. Are you male or female?**

| □ male | □ female |
| --- | --- |

**22. How old are you?**

**23. Are you currently married?**

| □ married | □ not married |
| --- | --- |

**24. Do you live by yourself or with anyone else?**

| □ live by myself | □ live with others |
| --- | --- |

**25. What is your postcode? (this information will**

**not identify you, we would just like to check that**

**questionnaires come from across the UK)**

**26. How many years of education have you received?**

**(e.g. if you attended school from the ages of 5 - 16 years,**

**you have received 12 years of education)**

**27. What is your ethnic group?**

**28. Do you smoke?**

□ I have never smoked

□ I used to smoke, but no longer smoke

□ On average, I smoke less than 2 cigarettes or equivalent per day

□ On average, I smoke 2 - 10 cigarettes or equivalent per day

□ On average, I smoke more than 10 cigarettes or equivalent per day

**29. Do you take vitamin or mineral supplements?**

□ I never take vitamin or mineral supplements

□ I take vitamin or mineral supplements occasionally

□ I take vitamin or mineral supplements regularly

**30. Do you drink alcohol? (1 unit of alcohol is the equivalent of 1 half pint of beer or lager, 1 glass of wine, 1 measures of spirits)**

□ I never drink alcohol

□ I used to drink alcohol, but never drink alcohol now

□ I drink 1-4 units of alcohol per week

□ I drink 5-14 units of alcohol for women or 5-21 units of alcohol per week for men

□ I drink over 14 units of alcohol for women or 21 units of alcohol per week for men

**31. What is your height?**

| m / cm |  |
| --- | --- |

feet / inches

**32. What is your weight?**

kg

stone / lbs

**33. How much physical activity do you do? (please tick all those that apply)**

□ I do no physical activity

□ I stand up or walk around all day

□ I walk or cycle to work

□ I regularly undertake exercise or sport approx. 1-2 times per week

□ I regularly undertake exercise or sport approx. 3-5 times per week

□ I regularly undertake exercise or sport approx. 6 times per week or more

**Thank you very much for taking part.**

**FOOD AND NUTRITION SURVEY**

Please read the following instructions carefully before completing the questionnaire

1. Please answer ALL the questions
2. Select the answer that best applies to your situation and TICK the appropriate column. Only tick ONE box on each line (ie. per food). If you make a mistake cross through the incorrect tick and tick the correct answer.

*HOW OFTEN DO YOU EAT EACH OF THE FOLLOWING FOODS?* **Base your answers on the last week (tick the appropriate box)**

**How often do you eat the following foods?** 2 or

more 3-5 1-2 1-3

times Every times times times Rarely/ a day day a week a week a month never

**DAIRY PRODUCTS**

Milk (including in tea and coffee: Whole 🞏 🞏 🞏 🞏 🞏 🞏

Semi-skimmed 🞏 🞏 🞏 🞏 🞏 🞏

Skimmed 🞏 🞏 🞏 🞏 🞏 🞏

Butter 🞏 🞏 🞏 🞏 🞏 🞏

Margarine (e.g. Stork, Clover) 🞏 🞏 🞏 🞏 🞏 🞏

Polyunsaturated Margarine 🞏 🞏 🞏 🞏 🞏 🞏

Low fat spreads (e.g. Outline, Gold) 🞏 🞏 🞏 🞏 🞏 🞏

Ice cream 🞏 🞏 🞏 🞏 🞏 🞏

Yoghurt, fromage frais 🞏 🞏 🞏 🞏 🞏 🞏

Cheese (e.g. cheddar, stilton, cream cheese) 🞏 🞏 🞏 🞏 🞏 🞏

Low fat Cheese (e.g. cottage, reduced fat) 🞏 🞏 🞏 🞏 🞏 🞏

Eggs – fried 🞏 🞏 🞏 🞏 🞏 🞏

- not fried (boiled, poached, in baking) 🞏 🞏 🞏 🞏 🞏 🞏

Cheese and/or egg dishes 🞏 🞏 🞏 🞏 🞏 🞏

(e.g. pizza, quiche, macaroni cheese)

**MEATS AND FISH**

Beef – roast/steak 🞏 🞏 🞏 🞏 🞏 🞏

Lamb – roast/chops 🞏 🞏 🞏 🞏 🞏 🞏

Pork – roast/chops 🞏 🞏 🞏 🞏 🞏 🞏

Chicken, turkey or other poultry 🞏 🞏 🞏 🞏 🞏 🞏

Bacon or gammon 🞏 🞏 🞏 🞏 🞏 🞏

Meat dishes (e.g. stew, curry, chilli, lasagne) 🞏 🞏 🞏 🞏 🞏 🞏

Canned meats (e.g. corned beef, ham) 🞏 🞏 🞏 🞏 🞏 🞏

Meat pies, sausage rolls or pasties 🞏 🞏 🞏 🞏 🞏 🞏

Sausages or beefburgers 🞏 🞏 🞏 🞏 🞏 🞏

Liver, kidney, pate (other offal products) 🞏 🞏 🞏 🞏 🞏 🞏

Fish and seafood - not fried 🞏 🞏 🞏 🞏 🞏 🞏

- fried 🞏 🞏 🞏 🞏 🞏 🞏

- canned (e.g. tuna) 🞏 🞏 🞏 🞏 🞏 🞏

**BREAD**

White bread 🞏 🞏 🞏 🞏 🞏 🞏

Brown / granary bread 🞏 🞏 🞏 🞏 🞏 🞏

Wholemeal bread (inc. chapattis) 🞏 🞏 🞏 🞏 🞏 🞏

**How often do you eat the following foods?** 2 or

more 3-5 1-2 1-3

times Every times times times Rarely/

a day day a week a week a month never

**CEREALS**

Sweet Biscuits (plain and chocolate) 🞏 🞏 🞏 🞏 🞏 🞏

Crackers / crispbread 🞏 🞏 🞏 🞏 🞏 🞏

Cakes, buns, pastries 🞏 🞏 🞏 🞏 🞏 🞏

Puddings (e.g. fruit pies, cheesecake) 🞏 🞏 🞏 🞏 🞏 🞏

Breakfast Cereal:

High fibre (e.g. bran flakes, Weetabix) 🞏 🞏 🞏 🞏 🞏 🞏

Ordinary (e.g. cornflakes, rice krispies) 🞏 🞏 🞏 🞏 🞏 🞏

Sweet (e.g. coco pops, frosties) 🞏 🞏 🞏 🞏 🞏 🞏

Muesli 🞏 🞏 🞏 🞏 🞏 🞏

Rice or pasta 🞏 🞏 🞏 🞏 🞏 🞏

**FRUIT ANE VEGETABLES**

Apples, pears 🞏 🞏 🞏 🞏 🞏 🞏

Oranges, grapefruit or other citrus fruit 🞏 🞏 🞏 🞏 🞏 🞏

Bananas 🞏 🞏 🞏 🞏 🞏 🞏

Green vegetables (e.g. cabbage, peas, broccoli) 🞏 🞏 🞏 🞏 🞏 🞏

Carrots, tomatoes (fresh or canned) 🞏 🞏 🞏 🞏 🞏 🞏

Other vegetables (including salad items) 🞏 🞏 🞏 🞏 🞏 🞏

Baked beans 🞏 🞏 🞏 🞏 🞏 🞏

Other beans / lentils / dahl 🞏 🞏 🞏 🞏 🞏 🞏

Vegetable dishes (e.g. stew, curry – NO meat) 🞏 🞏 🞏 🞏 🞏 🞏

Potatoes – chips, roasted, fried 🞏 🞏 🞏 🞏 🞏 🞏

- not fried (boiled, baked) 🞏 🞏 🞏 🞏 🞏 🞏

**BEVERAGES**

Beer or lager 🞏 🞏 🞏 🞏 🞏 🞏

Wine, sherry or spirits 🞏 🞏 🞏 🞏 🞏 🞏

Tea or coffee 🞏 🞏 🞏 🞏 🞏 🞏

Squash 🞏 🞏 🞏 🞏 🞏 🞏

Fizzy or high energy drinks (Lucozade, red bull) 🞏 🞏 🞏 🞏 🞏 🞏

Low calorie drinks 🞏 🞏 🞏 🞏 🞏 🞏

Pure fruit juices 🞏 🞏 🞏 🞏 🞏 🞏

**MISCELLANEOUS**

Chocolate 🞏 🞏 🞏 🞏 🞏 🞏

Sweets 🞏 🞏 🞏 🞏 🞏 🞏

Sweet spreads (e.g. jam, marmalade) 🞏 🞏 🞏 🞏 🞏 🞏

Sugar (e.g. in tea/coffee, on cereal) 🞏 🞏 🞏 🞏 🞏 🞏

Crisps and savoury snacks 🞏 🞏 🞏 🞏 🞏 🞏

Nuts (including peanut butter) 🞏 🞏 🞏 🞏 🞏 🞏

Sauces and pickles (e.g. ketchup) 🞏 🞏 🞏 🞏 🞏 🞏

Salad oils, dressings and mayonnaise 🞏 🞏 🞏 🞏 🞏 🞏

**Are there any other foods that have not been recorded that you regularly eat?**

**Please write what they are and how often you eat them**

____________________________ 🞏 🞏 🞏 🞏 🞏 🞏

­­­­­­­­­­­­­­­­

____________________________ 🞏 🞏 🞏 🞏 🞏 🞏
